# Overexpression of *Vitreoscilla* hemoglobin increases waterlogging tolerance in *Arabidopsis* and maize

**DOI:** 10.1186/s12870-016-0728-1

**Published:** 2016-02-01

**Authors:** Hewei Du, Xiaomeng Shen, Yiqin Huang, Min Huang, Zuxin Zhang

**Affiliations:** Engineering research center of Ecology and Agricultural Use of wetland, Ministry of Education, Yangzte University, Jingzhou, 434025 P.R. China; National Key Laboratory of Crop Genetic Improvement, Huazhong Agricultural University, Wuhan, 430070 P.R. China; College of Life Science, Yangtze University, Jingzhou, Hubei 434025 P.R. China; Hubei Collaborative Innovation Center for Grain Crops, Yangtze University, Jingzhou, 434025 P.R. China; Food Crops Institute, Hubei Academy of Agriculture Sciences, Wuhan, 430064 P.R. China

**Keywords:** *Arabidopsis thaliana*, Maize (*Zea mays* L.), *Vitreoscilla* hemoglobin, Waterlogging, Genetic transformation

## Abstract

**Background:**

*Vitreoscilla* hemoglobin (VHb) is a type of hemoglobin found in the Gram-negative aerobic bacterium *Vitreoscilla* that has been shown to contribute to the tolerance of anaerobic stress in multiple plant species. Maize (*Zea mays* L.) is susceptible to waterlogging, causing significant yield loss. In this study, we approached this problem with the introduction of an exogenous *VHb* gene.

**Results:**

We overexpressed the *VHb* gene in *Arabidopsis* and maize under the control of the CaMV35S promoter. After 14 days of waterlogging treatment, the transgenic *VHb Arabidopsis* plants remained green, while the controls died. Under waterlogging, important plant growth traits of *VHb* plants, including seedling height, primary root length, lateral root number, and shoot dry weight were significantly improved relative to those of the controls. The *VHb* gene was also introduced into a maize line through particle bombardment and was then transferred to two elite maize inbred lines through marker-assisted backcrossing. The introduction of *VHb* significantly enhanced plant growth under waterlogging stress on traits, including seedling height, primary root length, lateral root number, root dry weight, and shoot dry weight, in both Zheng58 and CML50 maize backgrounds. Under the waterlogging condition, transgenic *VHb* maize seedlings exhibited elevated expression of alcohol dehydrogenase (*ADH1*) and higher peroxidase (POD) enzyme activity. The two *VHb-*containing lines, Zheng58 (VHb) and CML50 (VHb), exhibited higher tolerance to waterlogging than their negative control lines (Zheng58 and CML50).

**Conclusions:**

These results demonstrate that the exogenous *VHb* gene confers waterlogging tolerance to the transgenic maize line. In Maize in the place of to the transgenic maize line, the *VHb* gene is a useful molecular tool for the improvement of waterlogging and submergence-tolerance.

**Electronic supplementary material:**

The online version of this article (doi:10.1186/s12870-016-0728-1) contains supplementary material, which is available to authorized users.

## Background

*Vitreoscilla* hemoglobin (VHb) is one of the best understood bacterial hemoglobins. The VHb protein is a soluble hemoprotein containing two identical subunits, with a relative molecular mass of 15.8 kDα and two b hemes per molecule [1]. VHb is a single-domain hemoglobin possessing a similar structure as vertebrate globins [1]. The *VHb* gene has been expressed in various heterologous hosts, including bacteria [2], yeast [3], fungi [4], plants [5], and animals [6], and has been shown to improve growth and productivity under oxygen-limited conditions [7]. When *VHb* was expressed in *P. pastoris* under control of a methanol-inducible promoter, it enhanced the oxygen uptake rate and promoted methanol metabolism, thereby improving cell performance and β-galactosidase production [8]. When the *VHb* gene was expressed in zebrafish using the common carp β-actin promoter, the transgenic *VHb* zebrafish exhibited higher tolerance to hypoxia stress and a higher survival rate than the controls [6].

In addition, the *VHb* gene has been used in plants to improve waterlogging tolerance and productivity. When a CaMV35S*-*driven *VHb* gene was transferred into *Astragalus membranaceus* via *Agrobacterium rhizogenes*, the dry weight and growth rate of the hairy roots of transgenic *Astragalus membranaceus* were significantly higher than the controls, and the astragaloside IV content in the transgenic hairy roots was 5 to 6 times higher than that in the non-transgenic hairy root controls [9]. In transgenic *VHb* cabbage, seeds germinated faster than the wild-type controls, and the transgenic plants also showed tolerance to prolonged submergence [10]. *Arabidopsis* plants expressing exogenous *VHb* also exhibited an increased germination rate and improved submergence tolerance [11]. When transgenic petunias expressing *VHb* were submerged in liquid Murashige and Skoog (MS) media, they survived in the hypoxic conditions and grew out of the water surface, while the control plants did not. Thus, *VHb* transgenic petunias exhibited higher tolerance to submergence [5].

Waterlogging is a serious agricultural problem in many areas of the world [12]. These previous studies suggest that the *VHb* gene may serve as a useful tool for the improvement of plant tolerance to waterlogging and submergence, which cause oxygen deficiency in plant roots. Maize is an important crop, for which waterlogging has increasingly become a major constraint to its production in tropical and subtropical regions [13]. In this study, we expressed the *VHb* gene in *Arabidopsis* and maize under the control of the CaMV35S promoter and tested its effects on tolerance to waterlogging. Our results demonstrate that the expression of exogenous *VHb* in *Arabidopsis* and maize can significantly improve the tolerance of transgenic plants to waterlogging.

## Results

### *Arabidopsis* plants expressing exogenous *VHb* exhibit higher tolerance to waterlogging

**Fig. 1:**
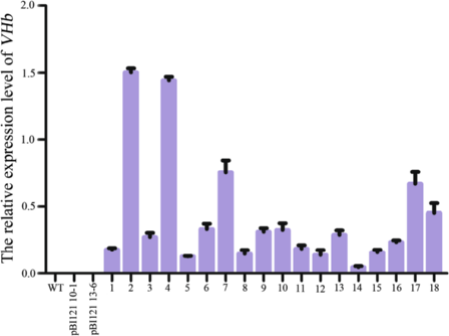
Relative *VHb* expression levels in leaves of different transgenic *Arabidopsis* lines. Total RNA samples were isolated from 30-day-old transgenic *VHb Arabidopsis* plants, reverse-transcribed into cDNA, and used for real-time qRT-PCR. The relative transcript levels were calculated using the *Arabidopsis actin1* gene (GenBank: NM179953) as the internal reference. The results represent the mean values ± SD of three independent analyses

We obtained 18 transgenic *VHb Arabidopsis* plantlets and 14 transgenic control plantlets containing the pBI121 empty vector that grew green leaves and well-developed roots from the kanamycin-selective MS medium (data not shown). To assess the expression levels of the *VHb* gene, quantitative real-time RT-PCR (qRT-PCR) experiments were performed; the results showed different *VHb* expression levels in the 18 transgenic *VHb Arabidopsis* lines, with lines #2, #4, #7, #17, and #18 displaying higher *VHb* levels than the others (Fig. 1).

**Fig. 2:**
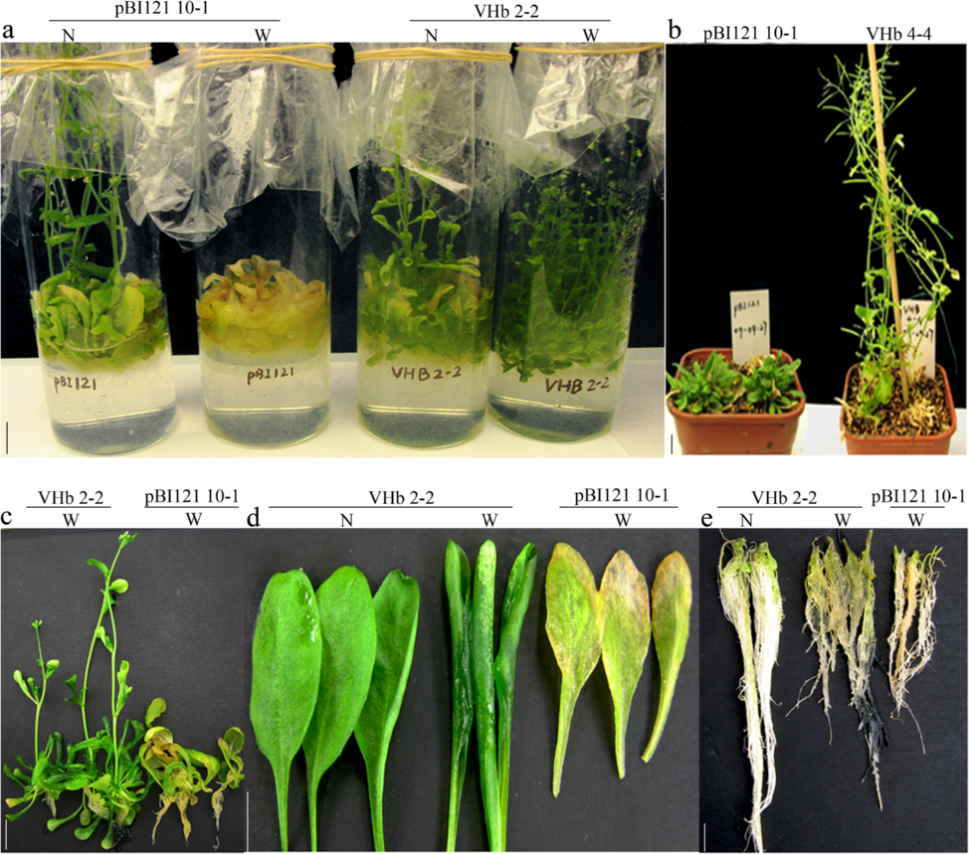
The appearance of transgenic *VHb Arabidopsis* after 14-day waterlogging stress treatment. Fourteen-day old plants grown in the tube were subjected to waterlogged (W) conditions or were untreated under normal (N) conditions for 14 days. **a**, Plants under 14-days of waterlogging stress or normal conditions. **b**, Plant development following waterlogging for 14 days and recovery for 14 days. **c**, Transgenic plants containing the *VHb* gene or the pBI121 empty vector subjected to waterlogging for 14 days. Plants were transferred from tubes to pots afterwards. **d**, Leaves of *VHb* and control plants after 14 days of waterlogging or without the treatment. **e**, Roots of *VHb* and control plants after 14 days of waterlogging or without the treatment. Two transgenic *VHb* lines (#2-2, and 4–4) and two empty vector control lines (#10-1 and 13–6); 20 seedlings for each line were subjected to waterlogging, and the experiment was repeated two times. A total of 60 seedlings for each line were used for waterlogging and another 60 were used as controls under normal conditions. Bar = 1 cm

Fourteen-day-old *Arabidopsis* plants grown in a tube containing 1/2 MS medium were subjected to waterlogging treatment for 14 days. During waterlogging, the transgenic *VHb* plants completely grew out of the water surface and continued to develop, while the control plants remained under water and were essentially arrested in plant development (Fig. 2a, c). The leaves of the *VHb* plants became curly during waterlogging and remained green. On the contrary, the leaves of the controls were fully expanded and turned yellow under waterlogging (Fig. 2d). The shoots and roots of transgenic *VHb* seedlings remained healthier than the controls during waterlogging (Fig. 2c, e), suggesting that transgenic *VHb* seedlings are more tolerant to waterlogging stress compared with the controls.

**Fig. 3:**
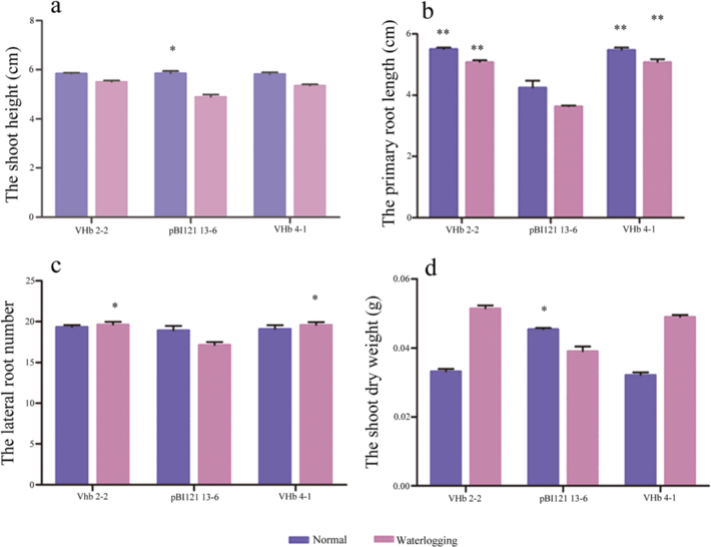
Growth traits of *VHb Arabidopsis* seedlings and controls under waterlogged or normal conditions for 14 days. **a**, Seedling height. **b**, Primary root length. **c**, Number of lateral roots. **d**, Shoot dry weight. Twenty seedlings for each line were waterlogged, and the experiment was repeated two times. Student’s *t*-test was performed. * indicates *p* < 0.05; ** indicates *p* <0.01

After 14 days of waterlogging stress, the average shoot height of the empty pBI121 vector plants was approximately 4.9 cm for line #13-6, which was significantly shorter than those that were untreated (averaged 5.9 cm) (Fig. 3a). Although the shoot height of the *VHb* plants also decreased under waterlogging (5.5 cm for line #2-2 and 5.4 cm for line #4-1) compared with the untreated *VHb* plants (approximately 5.8 cm), they were still taller than the waterlogged empty vector controls (4.9 cm) (Fig. 3a). The primary roots were also significantly longer (approximately 1.5 cm longer) than the empty vector controls both in waterlogged and untreated conditions, suggesting that the growth of primary roots is enhanced with expression of exogenous *VHb* (Fig. 3b). The *VHb* plants exhibited more lateral roots than the empty vector controls under waterlogging (Fig. 3c). Furthermore, after waterlogging for 14 days, the average shoot dry weight of the *VHb* plants was more than 20 % higher than that of the empty vector controls (Fig. 3d), suggesting better shoot growth under waterlogged conditions; however, the shoot dry weights of the *VHb* plants were lower than the controls under unstressed conditions. After 14 days of recovery following 14 days of waterlogging, the *VHb* plants blossomed and produced siliques normally, while the flowering time of the control plants was severely delayed (Fig. 2b).

### The *VHb* gene was introduced into maize inbred lines Zheng58 and CML50

**Fig. 4:**
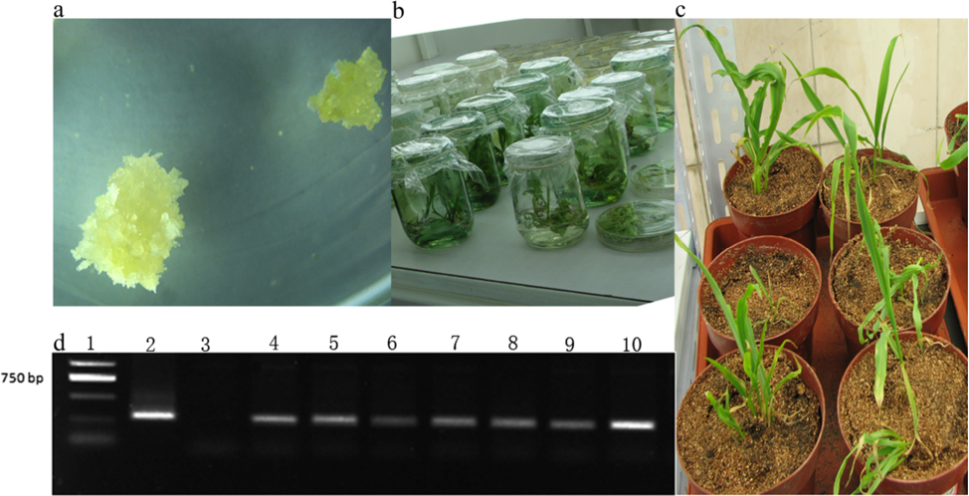
Generation and confirmation of transgenic *VHb* maize plants. **a**, Resistant calli. **b**, Plantlets regenerated from resistant calli. **c**, Plantlets grown in greenhouse. **d**, Genotyping for the transgene. PCR reactions were performed on the genomic DNA of putative transgenic maize plants using primers targeting the *VHb* gene. Lane 1, DL2000 DNA marker. Lane 2, positive control: the pCAMBIA3301-VHb construct. Lane 3, negative control: maize inbred line A188. Lanes 4–10, transgenic plants

**Fig. 5:**
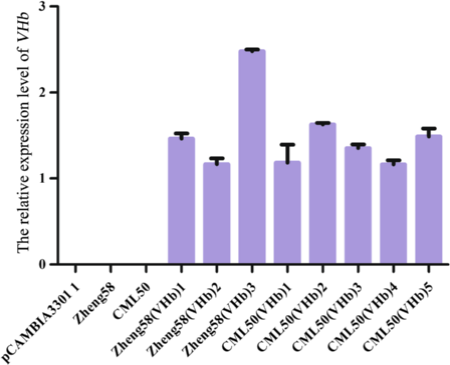
Relative *VHb* expression in *VHb* maize seedlings. Total RNA samples were isolated from the leaves of transgenic Zheng58 (VHb) and CML50 (VHb) seedlings, reverse-transcribed into cDNA, and used for real-time qRT-PCR. The relative expression levels of *VHb* were calculated by using the maize *actin1* gene (GRMZM2G126010) as the internal reference. The results represent the mean values ± SD of three independent analyses

Our results show that transgenic *VHb Arabidopsis* plants are more tolerant to waterlogging stress. As maize is one of the most important crops but is sensitive to waterlogging, submergence, and flooding, we applied the same approach to improve its tolerance to waterlogging. We transformed the *VHb* gene into maize by particle bombardment. Resistant calli were obtained after bialaphos selection (Fig. 4a); plantlets then emerged from the calli (Fig. 4b). Plantlets with well-developed roots were transferred into a growth chamber (Fig. 4c). PCR was performed to genotype the plantlets and the results confirmed that *VHb* was integrated into the maize chromosome (Fig. 4d). Subsequently, positive T_0_*VHb* plants were crossed with the popular maize inbred lines, Zheng58 and CML50. Three transgenic Zheng58 (VHb) and five CML50 (VHb) lines were obtained through molecular marker-assisted selection after six back crosses followed by one self-pollination. When the mRNA level of exogenous *VHb* in maize was determined using qRT-PCR, high *VHb* transcript levels were observed in all lines in both Zheng58 (VHb) and CML50 (VHb) genetic backgrounds (Fig. 5).

### The *VHb* gene leads to increased shoot height, primary root length, and the number of lateral roots in transgenic maize plants when placed under waterlogging stress

**Fig. 6:**
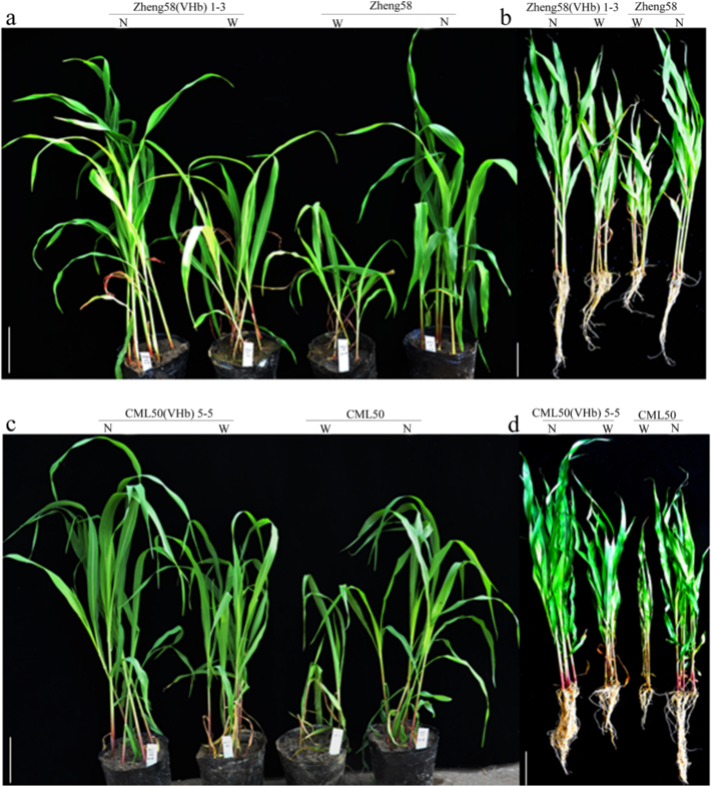
Growth traits of transgenic *VHb* maize plants and WT controls under waterlogged or normal conditions. **a-d**, plants under 7-days of waterlogging stress or normal conditions. N, normal condition; W, waterlogging stress for 7 days; bar = 10 cm

To test the tolerance to waterlogging, seedlings with three visible leaves from the transgenic maize lines were subjected to waterlogging for 7 days. The results showed that the transgenic *VHb* plants in the two genetic backgrounds grew stronger than wild-type (WT) plants during waterlogging stress (Fig. 6). Therefore, exogenous *VHb* expression also conferred waterlogging tolerance to maize plants.

**Fig. 7:**
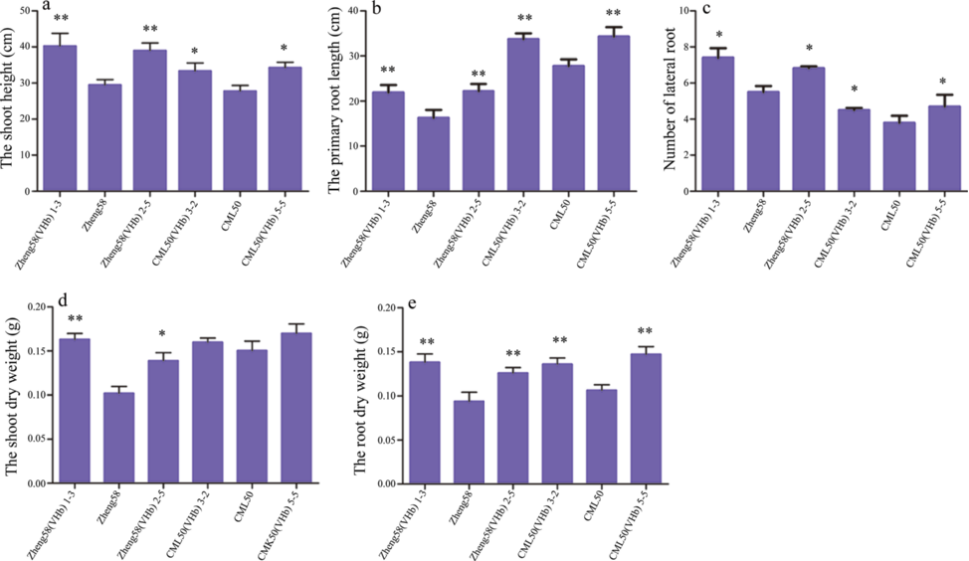
Differences in plant growth traits between transgenic *VHb* and wild-type plants under waterlogging stress. **a**, Seedling height. **b**, Primary root length. **c**, Number of lateral roots. **d**, Shoot dry weight. **e**, Root dry weight. Eighteen seedlings at the three-leaf stage were subjected to waterlogging stress; three replications were performed. After 7 days of waterlogging, traits including seedling height, primary root length, and the number of lateral roots were measured. After measurement, shoots and roots were placed in an oven (65 °C) for 3 days; the weight of the shoots and roots were then measured. The results (in cm or g) are represented by the mean values ± SD of three independent analyses. Student’s *t*-test was performed to reveal the significance between transgenic *VHb* maize and WT controls under waterlogged conditions. * indicates *p* < 0.05; ** indicates *p* <0.01

Under normal conditions, seedlings expressing *VHb* grew faster than the controls. The shoot height of Zheng58 (VHb)1-3 (40.18 cm), Zheng58 (VHb)2-5 (39.23 cm), CML50 (VHb)3-2 (39.13 cm), and CML50 (VHb)5-5 (39.18 cm) was significantly taller than the controls Zheng58 (35.67 cm) and CML50 (36.99 cm) (Additional file 1: Figure S1a). These results indicate that the expression of the exogenous *VHb* gene in maize can promote plant growth of Zheng58 and CML50. Under waterlogging stress, the growth of seedlings was inhibited in the absence of *VHb*, resulting in shorter Zheng58 (29.51 cm) and CML50 (27.76 cm) plants compared with untreated WT plants (Fig. 7a; Additional file 1: Figure S1a). However, waterlogged transgenic maize Zheng58 (VHb)1-3, Zheng58 (VHb)2-5, CML50 (VHb)3-2, and CML50 (VHb)5-5 exhibited similar seedling heights as untreated transgenic plants (Fig. 7a; Additional file 1: Figure S1a), suggesting that the *VHb* gene improves waterlogging tolerance in the two inbred lines.

Additionally, under normal conditions, the average primary root lengths of transgenic plants Zheng58 (VHb)1-3 (32.54 cm), Zheng58 (VHb)2-5 (34.28 cm), CML50 (VHb)3-2 (38.01 cm), and CML50 (VHb)5-5 (39.18 cm) were not significantly different from those of non-transgenic Zheng58 (37.40 cm) and CML50 (36.98 cm) plants (Additional file 1: Figure S1b). However, under waterlogged conditions, the lengths of the primary roots of Zheng58 (VHb)1-3 (21.91 cm), Zheng58 (VHb)2-5 (22.21 cm), CML50 (VHb)3-2 (33.71 cm), and CML50 (VHb)5-5 (34.29 cm) were significantly longer than those of the corresponding waterlogged WT without the *VHb* gene (Fig. 7b). These results indicate that the introduction of the *VHb* gene might alleviate the waterlogging-induced repression of primary roots elongation in maize.

Under untreated conditions, the numbers of lateral roots of WT lines and transgenic *VHb* lines were not significantly different (Additional file 1: Figure S1c). However, when subjected to waterlogging, the lateral roots were induced to emerge in the *VHb* transgenic lines. Transgenic lines Zheng58 (VHb)1-3 (7.4 on average), Zheng58 (VHb)2-5 (6.8 on average), CML50 (VHb)3-2 (4.5 on average), and CML50 (VHb)5-5 (4.7 on average) grew more lateral roots compared with their WT lines, Zheng58 (5.4 on average) and CML50 (3.8 on average) (Fig. 7c). These results indicate that transgenic seedlings might achieve tolerance to waterlogging by enhancing the growth of lateral roots.

### The presence of *VHb* increases the dry weights of shoots and roots in maize under waterlogging

The shoot dry weight of maize plants is sensitive to waterlogging stress [14]. Under waterlogging, the shoot dry weight of Zheng58 was decreased. However, the shoot dry weights of waterlogged Zheng58 (VHb) 1–3 and 2–5 were significantly heavier than that of waterlogged Zheng58 plants (Fig. 7d), indicating that the *VHb* gene improves shoot growth under waterlogged condition. The root dry weights of CML50 and Zheng58 were greatly decreased when subject to waterlogging (Fig. 7e). The introduction of the *VHb* gene was able to alleviate the decrease in the root dry weight of the Zheng58 (VHb) and CML50 (VHb) lines by accumulating high levels of dry matter in the roots (Fig. 7e).

### *ADH1* expression and peroxidase enzyme activity are induced by the introduction of *VHb*

**Fig. 8:**
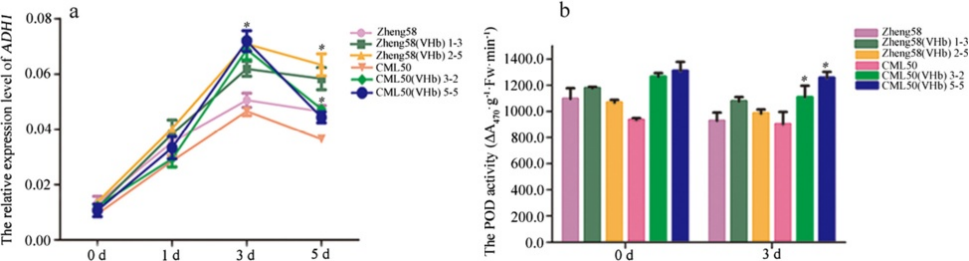
Induction of *ADH1* mRNA expression and POD activity in response to waterlogging. **a**, *ADH1* mRNA level. **b**, Peroxidase enzyme activity. Zheng58, Zheng58 (VHb), CML50, and CML50 (VHb) seedlings were subjected to waterlogging. Roots collected at different time points under waterlogged conditions were used to isolate total RNA and extract POD. The *ADH1* mRNA levels were determined by qRT-PCR. The peroxidase activity represents the mean values ± SE of three independent analyses. Student’s *t*-test was performed. * indicates *p* < 0.05; ** indicates *p* < 0.001

The expression levels of the alcohol dehydrogenase 1 (*ADH1*) gene in the roots at different time points under waterlogging stress were measured. The *ADH1* transcript levels in roots were induced from day 1 to day 5 when subjected to waterlogging and peaked at day 3. At days 3 and 5 under waterlogging, the *ADH1* transcript levels in Zheng58 (VHb) and CML50 (VHb) were significantly higher than those of their WT controls (Fig. 8a), indicating that the presence of the *VHb* gene in plants effectively induces the expression of anaerobic-stress related genes, such as *ADH1*, under waterlogged conditions. The peroxidase (POD) enzyme activity in the waterlogged roots was also analyzed. The results showed that the POD activity was higher in CML50 (VHb) seedlings than in WT controls under both waterlogging and untreated conditions (Fig. 8b). The POD activity was also increased in Zheng58 (VHb) relative to that in Zheng58, but this was not statistically significant. These results confirmed that seedlings with overexpression of *VHb* display enhanced expression of *ADH1* and higher POD activity.

## Discussion

Many QTLs associated with waterlogging tolerance at the seedling stage have been mapped in maize. For example, eleven root length-associated QTLs were detected in maize; among them, 10 QTLs were detected under waterlogging stress. In addition, fifteen QTLs for plant height, nine for shoot dry weight, one for root length, and four for root dry weight were also mapped [14]. Six of these QTLs were significantly associated with root dry weight under waterlogging stress in maize [15]. Three QTLs for primary root length and one for adventitious root weight were located on chromosome 4 of maize [16]. These reports suggest that plant growth traits, such as plant height, root length, shoot dry weight, and root dry weight, are tightly associated with waterlogging tolerance in maize and are thus good indicators of waterlogging tolerance.

In the present study, we chose plant growth traits, including shoot height, primary root length, lateral root number, shoot dry weight, and root dry weight, as the parameters to analyze waterlogging tolerance in *Arabidopsis* and maize. The seedling height of *Arabidopsis* plants with an empty pBI121 vector were significantly decreased under waterlogging but not for those of plants expressing *VHb*. Under waterlogged stress, the average length of the primary roots and the number of lateral roots in transgenic *VHb* plants were significantly greater than those in waterlogged control *Arabidopsis* plants. More importantly, the growth traits including the seedling height, primary root length, number of lateral roots, shoot dry weight, and root dry weight, were significantly increased in *VHb* maize under waterlogging stress in comparison with those of the WT maize controls without *VHb*. These traits are associated with waterlogging tolerance; consequently, seedling performance under waterlogging is significantly improved in both transgenic *VHb Arabidopsis* and maize, resulting in waterlogging tolerance. Therefore, the changes in seedling height, primary root length, number of lateral roots, shoot dry weight, and root dry weight in both *Arabidopsis* and maize are morphological adaptations caused by the *VHb* gene that lead to waterlogging tolerance.

Previous research showed that the *VHb* gene is capable of promoting respiratory activity and ATP production under hypoxic conditions [17] and has been applied to animals and plants to improve tolerance to hypoxia and submergence stresses. In animals, the *VHb* gene was shown to decrease the mortality rate of fish under hypoxia stress; the survival rate of transgenic *VHb* zebrafish is significantly greater than the controls under 2.5 % O_2_ [6]. In plants, heterologous *VHb* expression has successfully enhanced plant tolerance to submergence stress. For example, transgenic *VHb* cabbage and potato show tolerance to prolonged submergence stress [10,18]. Rice plants expressing *VHb* and the trans-zeatin secretion gene (*tzs*) display enhanced growth with a significant increase in plant height and panicle length [19]. The mechanism of *VHb* in conferring tolerance to waterlogging and hypoxia stress is still unclear. The phenotypic changes during waterlogging are the consequences of many adaptive modifications that lead to waterlogging tolerance. In this study, the seedling height, primary root length, number of lateral roots, and shoot dry weight in *VHb* seedlings were significantly increased during waterlogging in *Arabidopsis* and maize, as well as the root dry weight in maize. Transgenic *VHb Arabidopsis* and maize showed enhanced growth compared with their controls under waterlogged conditions. The dry matter accumulation in the shoots and roots of *VHb* seedlings was also greater than that of the controls under waterlogged conditions. The changes in plant growth and architecture likely contribute to waterlogging tolerance. In addition, the enhanced induction of detoxifying enzyme activity is another adaptive mechanism. Under limited oxygen conditions, reactive oxygen species (ROS) are induced in stressed cells and cause oxidative damage to cellular components [20]. The role of peroxidase is to scavenge ROS and protect plant cells from oxidative damage. Therefore, higher activity of peroxidase would result in lower ROS accumulation [21]. Previous studies showed that the VHb protein exhibits peroxidase activity and is capable of reacting with H_2_O_2_ to yield reactive intermediates [22]. VHb can act as an oxidase itself or enhance the function of cytochromes by scavenging ROS and NO [23,24], resulting in enhanced growth of the host under anaerobic conditions. In addition, under waterlogged conditions, the plant cells switch from oxidative respiration to fermentation glycolysis. *ADH1* expression is often induced in many plants under waterlogged conditions, allowing glycolysis to continue by pyruvate consumption and recycling of NAD^+^ [25]. Anoxia and submergence tolerance of plants is often associated with high levels of *ADH1* mRNA [26]. In the present study, the activity of peroxidase and the mRNA level of *ADH1* in transgenic *VHb* maize plants are higher than the controls under waterlogged conditions, suggesting that transgenic *VHb* maize plants have a stronger ability to remove the accumulated ROS and acetaldehyde, resulting in a higher tolerance to waterlogging.

The world population is expected to reach 9 billion in 2050, raising the demand for food. Maize, as one of the most important crops, is susceptible to waterlogging. Waterlogging is increasingly becoming one of the major constraints to maize production and productivity. For example, in Southeast Asia, approximately 15 % of all maize growing areas face waterlogging problems, resulting in 25-30 % yield loss annually [13]. Thus, addressing the waterlogging problem has become an urgent issue. In the present study, the *VHb* gene has been transferred into two elite maize inbred lines, Zheng58 and CML50. All of the transgenic *VHb* seedlings are more tolerant to waterlogging than their controls. Zheng58 is the female parent of Zhengdan958, a hybrid maize variety that is widely planted in China. CML50 is also an elite inbred line that is widely cultivated. The transgenic maize lines developed in this study can be readily employed as donors of the *VHb* gene. These lines will improve the waterlogging tolerance of hybrid maize and maintain maize production and productivity under waterlogged conditions. Therefore, our report represents a significant advancement in the improvement of waterlogging tolerance in maize.

## Conclusions

The *VHb* gene has been transformed into *Arabidopsis* and maize. Transgenic *VHb* seedlings of *Arabidopsis* and maize were subjected to waterlogging treatment and displayed enhanced waterlogging tolerance. We found changes in traits of plant growth in transgenic *VHb Arabidopsis* and maize under waterlogging stress, as well as in *ADH1* expression levels and peroxidase activity in maize. These changes in growth traits and detoxifying enzyme levels are associated with the introduction of the exogenous *VHb* gene, resulting in waterlogging tolerance. We conclude that the *VHb* gene is a useful tool for the improvement of waterlogging and submergence tolerance in crops.

## Methods

### Plant growth

*Arabidopsis* seeds were treated with 70 % ethanol for 1 min, soaked in 1 % sodium hypochlorite for 15 min, and rinsed with sterile water 4–5 times. Sterilized seeds were transferred to MS medium and grown in a chamber at 19-21 °C for 10 days. Ten-day-old seedlings were planted at a density of 2 plants per 25 cm^2^ in moistened potting soil covered with a nylon window screen. Seedlings were grown in the greenhouse at 19-21 °C in a 16 h/ 8 h light–dark cycle.

Seeds of maize were germinated for 2 days in the dark on moist filter paper at room temperature. Germinated seeds were transferred to silica sand. Uniform seedlings with three visible leaves were subjected to waterlogging treatment.

### Binary construct and *Agrobacterium* strain

The full coding sequence (CDS) of the *VHb* gene (GenBank: AY278220) encoding 146 amino acids (aa) derived from *Vitreoscilla stercoraria* was isolated by RT-PCR and transferred into the pBI121 and pCAMBIA3301 binary vectors, respectively, both under control of the CaMV35S promoter. The pBI121 vector contains a Nos promoter-*NPT II* gene cassette and the pCAMBIA3301 vector contains a CaMV35S promoter-Bar gene cassette for the selection of transformants. Partial maps of the resulting constructs are shown in addition file 2 (Additional file 2: Figure S2). The pBI121-VHb construct and the empty pBI121 vector were mobilized individually into *Agrobacterium* GV3101 by a direct DNA transfer method [27]; and its integrity in *Agrobacterium* cells was confirmed by restriction enzyme analysis after extraction of the plasmid from GV3101.

### *Arabidopsis* and maize transformation

*Arabidopsis* transformation was carried out using the floral dip method according to Clough and Bent [28]. Mature seeds were harvested, dried down, and stored until selection. Seeds were treated with 70 % ethanol for 1 min, 1 % sodium hypochlorite for 15 min, followed by five rinses with sterile water. Sterilized seeds were planted on kanamycin selection plates (75 μg mL^−1^) and grown in a chamber at 21 °C under 16 h of lighting (50–100 μ Einsteins m^−2^s^−1^) for 10 days. Kanamycin-resistant seedlings with green leaves and well-established roots were selected from the plates and planted into moistened potting soil.

The maize calli derived from immature Hi-II zygotic embryos were used as target tissues for bombardment. The bombardment and subsequent selection of bar-resistant calli were performed according to Songstad [29]. Briefly, type II calli derived from Hi-II were transferred to osmotic medium and then bombarded two times. Bombarded calli were incubated at 28 °C in the dark. After selection and regeneration, plantlets grew out from bar-resistant calli. The genomic DNA of putative transgenic maize plantlets was isolated, and PCR was performed to confirm the presence of the transgene (Table 1). The T_0_ transgenic *VHb* plants were used as the donors, and two maize inbred lines (Zheng58 and CML50) were used as recurrent parents. After six cycles of marker-aided backcrossing and one instance of self-pollination, we obtained three homozygous Zheng58 (VHb) and five homozygous CML50 (VHb) lines. These transgenic lines were named Zheng58 (VHb) 1 to 3, and CML50 (VHb) 1 to 5. The expression levels of *VHb* in these homozygous lines were determined by qRT-PCR. In the next generation, the lines including Zheng58 (VHb)1-3, Zheng58 (VHb)2-5, CML50 (VHb)3-2, and CML50 (VHb)5-5 that expressed high levels of *VHb* and produced enough seeds were chosen for further research.

**Table 1:** Primers used in this study

Primers	Sequence (5´-3´)	Usage
VHb c-forward	ATGTTAGACCAGCAAACCATTAA	RT-PCR for full-length CDS of *VHb*
VHb c-reverse	TTATTCAACCGCTTGAGCGTACA	RT-PCR for full-length CDS of *VHb*
VHb forward	CCAGCAAACCATCAACATCA	Amplifying the *VHb*gene
VHb reverse	CGTAAAGGTCAGCCTCAACC	Amplifying the *VHb* gene
VHb q-forward	GACTATCAACATCATCAAGGC	qRT-PCR for *VHb* gene
VHb q-reverse	GACATCAGCAATAACACCGTA	qRT-PCR for *VHb* gene
ADH1 forward	GTGGCTGTTTTCGGTTTAGGA	qRT-PCR for maize *ADH1* gene
ADH1 reverse	ACTGGCTTGTTGTGGTCTTTT	qRT-PCR for maize *ADH1* gene
Ara-Actin1 forward	CCCCTGCTATGTATGTGGCTAT	qRT-PCR for *ArabidopsisActin1* gene
Ara-Actin1 reverse	GACAATTTCACGCTCTGCTGT	qRT-PCR for *ArabidopsisActin1* gene
Maize-Actin1 forward	TGTTGCTATCCAGGCTGTTCT	qRT-PCR for maize *Actin1* gene
Maize-Actin1 reverse	TCATTAGGTGGTCGGTGAGGT	qRT-PCR for maize *Actin1* gene

### Waterlogging treatment

All waterlogging treatments were performed in three independent biological replicates. The transgenic *Arabidopsis* T_2_ lines with high *VHb* expression levels were chosen for waterlogging treatment. Sterilized seeds were grown in kanamycin-containing MS medium (75 μg mL^−1^ kanamycin) for 7 days and then transferred into a tube containing 1/2 MS medium. Each tube contained four plants. Waterlogging treatments with sterile water were performed on 14-day seedlings, where the plants were submerged in water with only 1/3 of the top leaf left in the air. The plants were kept in a growth chamber at 21 °C under 16 h of light (50–100 μ Einsteins m^−2^s^−^1) for 14 days. Ten tubes for every genotype were chosen. Five of the ten tubes were subjected to waterlogging treatment, and the others were used as controls for the untreated normal conditions.

The germinated seeds of Zheng58, CML50, and transgenic Zheng58 (VHb) and CML50 (VHb) were planted in silica sand pots (18 cm × 16 cm) and grown in a greenhouse. Each pot contained six seeds. At the three-leaf stage, three pots per genotype were subjected to waterlogging for 7 days, and three pots remained in the normal conditions. The wterlogging treatment was performed in a pool. The maize seedlings in pots were submerged 2 ~ 3 cm under the water surface. All of the waterlogging treatments for *Arabidopsis* and maize were performed in three independent biological replicates.

### Phenotypic analysis

After 14-days of waterlogging, a total of sixty seedlings, each of the transgenic *Arabidopsis* seedling lines VHb2-2, VHb4-1, pBI121 10–1, and pBI121 13–6, were chosen for the measurement of seedling height (cm), primary root length (cm), and the number of lateral roots. After measurement, the shoots were detached, and placed in an oven (65 °C) for 3 days. Subsequently, the shoot dry weight (g) was determined. These traits were also measured in transgenic *VHb* and control seedlings under normal conditions. After waterlogging treatment for 7 days, all of the aforementioned traits and root dry weight (g) were measured for Zheng58, Zheng58 (VHb), CML50, and CML50 (VHb) seedlings. A total of 54 seedlings for each line were measured, and the mean of each trait was calculated based on three biological replicates. Student’s *t*-test was performed to determine the significance in differences between transgenic *VHb* plants and controls for each trait under waterlogged conditions.

### *ADH1* expression and POD activity

Total RNA samples were isolated from roots collected at different time points during waterlogging using the Trizol reagent (Invitrogen, Carlsbad, CA, USA). First strand cDNA was synthesized using SuperScript-II reverse transcriptase according to the manufacturer’s instructions (Invitrogen). The *actin1* gene (Maizegdb: GRMZM2G126010) was used as an endogenous control to normalize the expression data (Table 1). The qRT-PCR primers specific for *ADH1* (Maizegdb: GRMZM2G442658) and *Actin1* were listed in Table 1. Real-time PCR was conducted using the SYBR real-time PCR kit (Takara Japan) with IQTM SYBR® Green Supermixture according to the manufacturer’s instructions (Bio-Rad USA). The reaction conditions were as follows: 94 °C for 1 min, followed by 40 cycles of 95 °C for 10 s, 55 °C for 10 s, and 72 °C for 15 s.

The peroxidase protein was extracted as described in Agostini et al [30]. Ground samples of root tissues (0.1 g) from waterlogged and untreated seedlings were resuspended (1 h, 4 °C) with 1.5 mL of extraction buffer. Samples were homogenized for 1 min and then centrifuged at 4 °C for 15 min. For each sample, 0.1 mL supernatant was taken to measure the peroxidase activity at 470 nm using a spectrophotometer.

### Availability of supporting data

All supporting data can be found within the manuscript and its additional files.

## Additional files

Additional file 1:
**Figure S1.** Differences in plant growth traits between transgenic *VHb* and wild-type plants under normal conditions. a. Seedling height, b. Primary root length, c. Number of lateral roots, d. Shoot dry weight, e. Root dry weight. Eighteen seedlings at the three-leaf stage had traits analysis performed; three replications were performed. These traits, such as seedling height, primary root length, and the number of lateral roots, were measured. After measurement, shoots and roots were placed in an oven (65 °C) for 3 days; the weight of the shoots and roots were then measured. The results (in cm or g) are shown as the mean values ± SD of three independent analyses. Student’s *t*-test was performed to reveal significance between transgenic *VHb* maize and their WT controls. * indicates *p* < 0.05; ** indicates *p* <0.01. (TIF 32513 kb) Additional file 2:
**Figure S2.** Schematic representation of plasmid construct. a, pBI121-*VHb*. b, pCAMBIA3301-*VHb*. Drawings are not to scale. LB and RB, T-DNA left and right borders, respectively; Pnos, nopaline synthase gene promoter; Tnos, nopaline synthase gene terminator; T35s, CaMV35S terminator; P35s: CaMV35S promoter; *NPT*II, neomycin phosphotransferase II; VHb:Vitreoscilla hemoglobin. (TIF 268 kb)

## Abbreviations

aa: amino acid
*ADH1*: alcohol dehydrogenase
ATP: adenosine triphosphate
CaMV35S: CaMV35S promoter
CDS: coding sequence
NAD: nicotinamide adenine dinucleotide
POD: peroxidase
qRT-PCR: quantitative real time RT-PCR
ROS: reactive oxygen species
*tzs*: trans-zeatin secretion gene
*VHb*: Vitreoscilla hemoglobin
