# Supplementary figures and images for: Overexpression of Vitreoscilla hemoglobin increases waterlogging tolerance in Arabidopsis and maize
Source: BMC Plant Biol. 2016 Feb 1;16:35. doi: 10.1186/s12870-016-0728-1 (PMC4736479; doi:10.1186/s12870-016-0728-1)

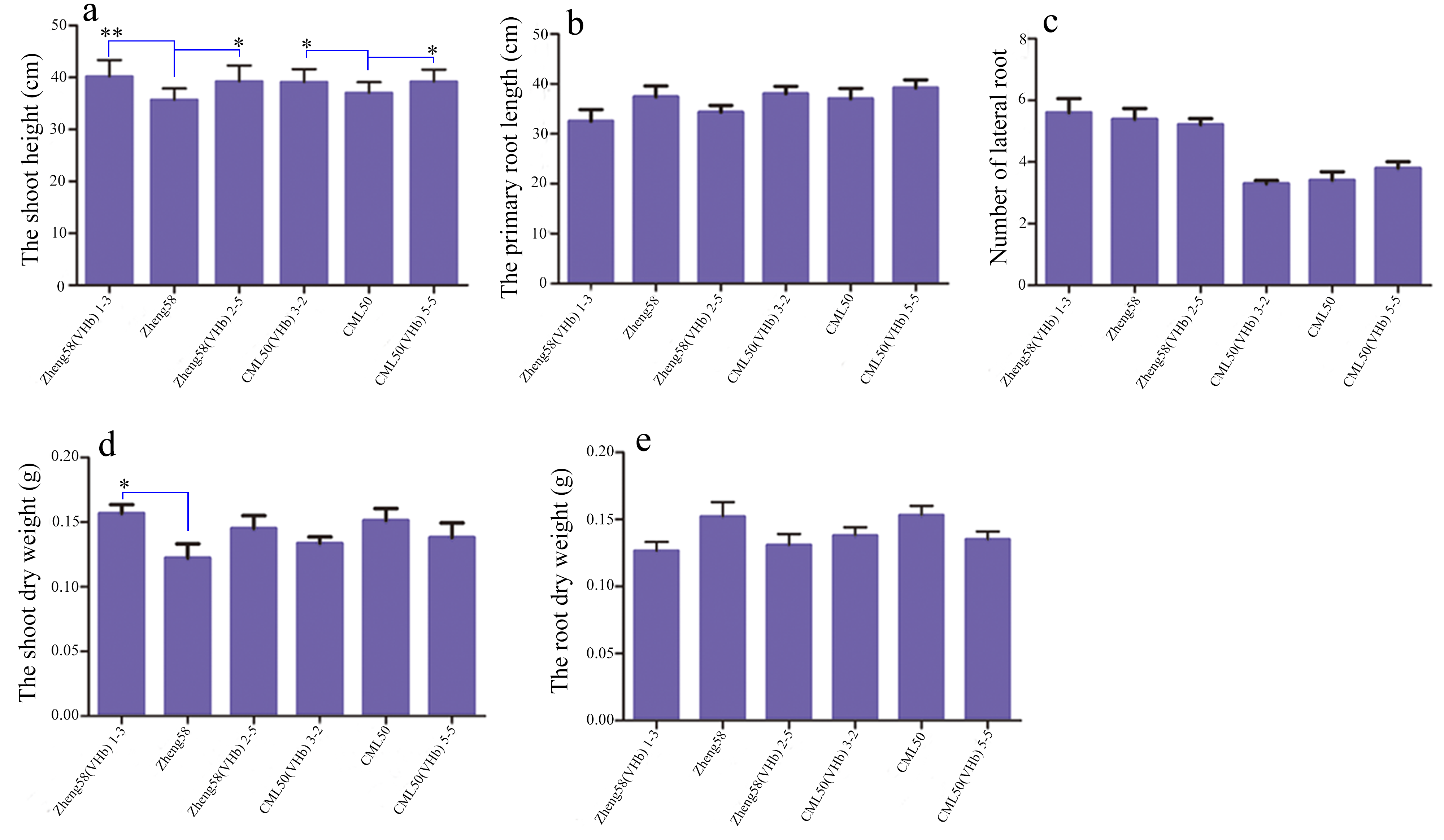

Supplement: Additional file 1: — Figure S1. Differences in plant growth traits between transgenic VHb and wild-type plants under normal conditions. a. Seedling height, b. Primary root length, c. Number of lateral roots, d. Shoot dry weight, e. Root dry weight. Eighteen seedlings at the three-leaf stage had traits analysis performed; three replications were performed. These traits, such as seedling height, primary root length, and the number of lateral roots, were measured. After measurement, shoots and roots were placed in an oven (65 °C) for 3 days; the weight of the shoots and roots were then measured. The results (in cm or g) are shown as the mean values ± SD of three independent analyses. Student’s t-test was performed to reveal significance between transgenic VHb maize and their WT controls. * indicates p < 0.05; ** indicates p <0.01. (TIF 32513 kb) [file 12870_2016_728_MOESM1_ESM.tif]

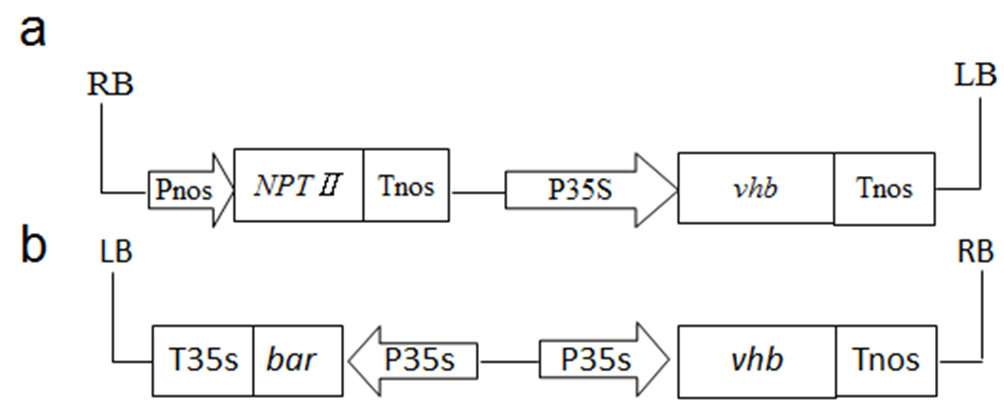

Supplement: Additional file 2: — Figure S2. Schematic representation of plasmid construct. a, pBI121-VHb. b, pCAMBIA3301-VHb. Drawings are not to scale. LB and RB, T-DNA left and right borders, respectively; Pnos, nopaline synthase gene promoter; Tnos, nopaline synthase gene terminator; T35s, CaMV35S terminator; P35s: CaMV35S promoter; NPTII, neomycin phosphotransferase II; VHb:Vitreoscilla hemoglobin. (TIF 268 kb) [file 12870_2016_728_MOESM2_ESM.tif]
